# Analysis of right heart flow patterns in repaired Tetralogy of Fallot with 4D flow-sensitive MRI

**DOI:** 10.1186/1532-429X-13-S1-P206

**Published:** 2011-02-02

**Authors:** Christopher J Francois, Shardha Srinivasan, Benjamin R Landgraf, Eric Niespodzany, Oliver Wieben, Alex Frydrychowicz

**Affiliations:** 1 University of Wisconsin, Madison, WI, USA

## Introduction

Cardiac MRI (CMR) is used to follow patients after TOF repair to assess pulmonary regurgitation (PR), pulmonary stenosis (PS) and right ventricular (RV) function. 4D flow-sensitive MRI techniques enable visualization of complex flow patterns [1],[2]. With the ability to simultaneously acquire morphology and hemodynamics for visualization and quantification, they may improve evaluation of functional outcomes following surgery for complex CHD.

## Purpose

Analyze flow patterns in superior vena cava (SVC), inferior vena cava (IVC), right atrium (RA), right ventricle (RV), and main, right and left pulmonary arteries (MPA, RPA, and LPA) using 3D radially-undersampled, 4D flow-sensitive MRI.

## Methods

This HIPAA-compliant study was performed in 11 patients with TOF (5M/6F; 20.1±12.4 years) on 1.5T and 3.0T clinical systems (GE Healthcare, Waukesha, WI) after IRB-approval and obtaining consent. 4D flow-sensitive MRI data were acquired with a radially undersampled phase contrast (PC) sequence, PC VIPR [2] with: 320mm^3^ volume, 1.0-1.25mm^3^ acquired isotropic spatial resolution, and VENC of 50-200cm/s. Scan time was approximately 8-12min using an adaptive respiratory bellows reading and 50% efficiency. Retrospective ECG-triggered cardiac gating was used with datasets reconstructed into 20 time frames. Flow visualization and analysis (Table 1) was performed using Ensight (CEI, Apex, NC) with particle trace emitter planes placed in SVC, IVC, tricuspid valve, and MPA**.**

**Table 1:** Grading system for evaluation of right heart flow patterns

SVC/IVC	1	S wave > D wave
	2	D wave > S wave
RA	1	normal, single clockwise vortex
	2	increased vortices
RV diastole	1	normal right-handed helix through TV
	2	increased helicity and vorticity
RV systole	1	uniform, laminar flow toward RVOT
	2	non-uniform outflow
MPA/RPA/LPA	1	uniform, laminar flow
	2	helical or vortical flow

## Results

SVC and IVC flow was greater during diastole than systole in 8/11. Increased RA vortices were present in 7/11. RV diastolic flow was normal in 1/11. RV systolic flow was normal in 9/11. MPA, RPA, and LPA flow was helical or vortical in 6/11, 10/11, and 10/11, respectively. PR and PS were present in 10/11 and 6/11, respectively.

## Conclusions

Flow patterns in the right heart of patients with repaired TOF are altered compared to previously reported flow patterns in normal subjects [3],[4]. These alterations may help explain the increased symptoms during exercise in the repaired heart. An additional benefit of acquiring 4D flow-sensitive MRI data is that flow analysis can be performed *post priori* through any area of interest.

**Figure 1:**
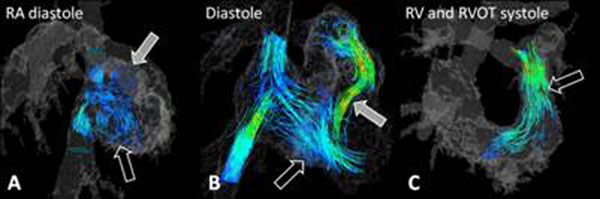
Right atrial and ventricular particle tracings. (A) A second RA vortex (closed arrow) was present during diastole in 7/11. (B) RV inflow was directed toward the RV apex (open arrow) in patients with PR (closed arrow). (C) RV outflow was normal in 9/11.
